# Principal component analysis is limited to low-resolution analysis in cryoEM

**DOI:** 10.1107/S2059798321002291

**Published:** 2021-05-19

**Authors:** Carlos Oscar S. Sorzano, Jose Maria Carazo

**Affiliations:** a National Center of Biotechnology (CSIC), Darwin 3, Campus Universidad Autónoma de Madrid, Cantoblanco, 28049 Madrid, Spain

**Keywords:** 3D reconstruction, image processing, single-particle cryoEM, structure determination, cryo-electron microscopy, macromolecular machines, protein structure

## Abstract

Principal component analysis (PCA) has been widely proposed to analyze flexibility and heterogeneity in cryo-electron microscopy (cryoEM). In this paper, it is argued that (i) PCA is an excellent technique to describe continuous flexibility at low resolution (but not so much at high resolution) and (ii) PCA components should be analyzed in a concerted manner (and not independently).

## Abstract 2

Principal component analysis can only give a low-frequency description of the movements that a macromolecule is undergoing.

## 1. Introduction   

Biological macromolecules can be regarded as flexible objects whose movements, which are continuous in a general case, allow them to perform their physiological functions. Principal component analysis (PCA) has been widely proposed to analyze flexibility and heterogeneity in cryo-electron microscopy (cryoEM) (Tagare *et al.*, 2015 ▸; Haselbach *et al.*, 2018 ▸; Punjani & Fleet, 2020 ▸). This technique assumes that the different conformations that are present in a sample can be constructed as a weighted sum of the eigenvectors of the covariance matrix of the volume. In its turn, the covariance has also been the subject of much previous work in the field (Penczek *et al.*, 2006 ▸; Zheng *et al.*, 2012 ▸; Andén *et al.*, 2015 ▸; Katsevich *et al.*, 2015 ▸; Liao *et al.*, 2015 ▸; Andén & Singer, 2018 ▸; Zhang *et al.*, 2019 ▸). In this paper, we argue that (i) PCA is an excellent technique to describe continuous flexibility at low resolution (but not so much at high resolution) and (ii) PCA components should be analyzed in a concerted manner (and not independently).

## 2. PCA coarsely describes movements   

Let 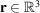 be an arbitrary location in space. Given an ideal conformation *V*_0_(**r**), any continuously deformed map *V*(**r**) could be constructed as 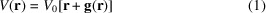
for some local, continuous deformation field 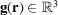. Note that every single particle would have its own **g**(**r**) deformation field different from the deformation fields of other particles. PCA approximates the deformed volume, more specifically the vector formed by its samples on a regular grid, **V**, by a linear combination of volumes, 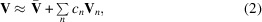
where 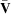 is the mean volume of all of the deformed volumes and **V**_*n*_ are the eigenvectors of the covariance matrix of the volumes (also called eigenvolumes). The approximation sign would be an equality if, for volumes of *N* ^3^ voxels, we compute *N* ^3^ eigenvolumes. With this formulation, PCA is more similar to a Taylor series expansion than to the continuous deformation field expressed above in (1), 
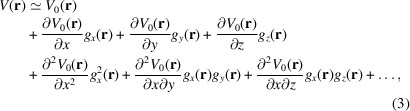
where *x*, *y* and *z* are the Cartesian directions. The first line is the undeformed volume, the second line comes from the Jacobian (first derivatives) of the undeformed volume, the third line comes from the Hessian (second derivatives) *etc*. Note that each of the terms of the Taylor expansion is a volume in itself. In this way, the Taylor expansion of a deformed volume is a sum of many volumes (each with less and less energy as long as the deformations are sufficiently small). The approximation error of the Taylor expansion decays as *o*[*∥* **g**(**r**)*∥* ^−*k*^], where *k* is the highest degree of the derivatives included in the sum.

Although PCA goes beyond the extreme locality of the Taylor expansion (because it has access to the whole population of observed data), the comparison here is relevant in the sense that it highlights the similarity in nature of the construction of a volume as the linear combination of a basis capturing different variations. PCA can also be interpreted as being related to factor analysis in a probabilistic generative model (Tipping & Bishop, 1999 ▸; Moghaddam, 2002 ▸).

An interesting remark is that constructing a basis for describing the movements of the coordinates of a volume (equation 1) is not the same as constructing a basis for describing a volume (equations 2 and 3). The second task requires many more terms than the first, as the small details, such as the atomic or secondary structure, require the addition of high-frequency terms. In contrast, in the first task these small details are provided by *V*(**r**), and **g**(**r**) only needs to explain their relative position with respect to the original location. In practice, slowly varying and small-amplitude **g**(**r**) deformations are expected. This is at the core of all deformable registration techniques between volumes in biomedical imaging (Sotiras *et al.*, 2013 ▸).

However, there is a fundamental difference between the Taylor expansion above and PCA: in the Taylor expansion, for each deformed particle the volumes to add are different, while in PCA the volumes to add are the same but with different weights, *c*_*n*_, that depend on each particle. From this point of view, we can regard PCA as a way to find a linear subspace that approximates all of the volumes used in the Taylor expansions of all of the particles in a data set; a way of performing a linear embedding of the manifold of volumes used in the Taylor expansion.

At this point, considerations of the signal-to-noise ratio of the images impose an intrinsic limit on the identifiability of PCA components in high-dimensional space (Johnstone & Paul, 2018 ▸), so that in a practical setting only a few PCA components can be explored/calculated from sets of cryoEM images, typically between one and three [apart from the work of Punjani & Fleet (2020 ▸), in all of the PCA applications we know of in cryoEM the number of PCA components analyzed is always smaller than three, although there is no special reason for such a low number].

Katsevich *et al.* (2015 ▸) provide an in-depth theoretical analysis of the properties of PCA in the context of cryoEM. It is shown that although the estimates of the covariance matrix are consistent for the number of images going to infinity, in common practice estimating the covariance matrix of a typical volume with a side width of 200–300 voxels would be prohibitively high. Combining the facts that PCA is a linear embedding of a potentially much more complex reality and that we can only access a quite small number of dimensions of that linear subspace, it is clear that PCA can only achieve a coarse description of the underlying deformed volumes.

PCA also has another exciting connection to harmonic functional analysis, highlighting its coarse representation of the underlying movements. As discussed below, we may think of PCA components as an *ad hoc* basis specifically tailored to describe sets of macromolecules. Indeed, PCA is equivalent to a Laplacian analysis of a graph in which all voxels are connected to all other voxels (He *et al.*, 2005 ▸) (which, in turn, is very much related to a dimensionality-reduction technique called locality-preserving projections; Sorzano *et al.*, 2014 ▸). The actual connectivity matrix is given by the covariance matrix in such a way that if the covariance between two voxels is large in absolute value then these two voxels are connected, and if it is low then these two voxels are not connected. We may compare this connectivity to that in which a voxel is only connected to its immediately neighboring voxels (and impose periodic conditions when we reach the edges of the bounding box). The eigenvectors of the Laplacian of the graph of this latter connectivity matrix happen to be the complex exponentials; that is, the basis used in the 3D Fourier transform (Saito, 2008 ▸). Actually, we may extend the concept of the Fourier transform to arbitrary geometrical shapes and construct a complete basis of functions defined in any arbitrary region by computing the eigenvectors of the Laplacian of its connectivity matrix (Saito, 2008 ▸; for instance, spherical harmonics is the resulting basis when we define the connectivity matrix of the surface of a sphere, Bessel functions are the basis for cylindrical surfaces and prolate spheroidals are the basis for solid spheres). In this regard, PCA would be in a superior position as it can identify coordinated movements between parts of the macromolecule that are not adjacent. The reason for this is that it has access to statistical information about the different macromolecule conformational states largely beyond the purely geometrical connectivity of adjacent voxels (note that the fact that the connectivity matrix is local does not constrain its analysis to local regions; for instance, the Fourier transform contains low-frequency components that can express long-range dependencies).

In Saito (2008 ▸) it is argued that the representation error (that is, how accurately the map is represented by a finite sum of elements of the basis) decays as *O*(*n* ^−α+0.5^) (where 1 < α < 2) when *n*, the number of elements in the truncated basis, goes to infinity (for example, for the Fourier transform α = 1). In this regard, the way that PCA is applied in cryoEM loses its theoretical advantage of having access to the correlation matrix. The reason for this is that we are normally restricted to no more than a few principal components, and consequently we must necessarily be incurring large representation errors due to the low-frequency nature of the components being used.

Either from the Taylor expansion argument or from the connection of PCA to harmonic functional analysis, we see that PCA can only describe very coarse (low-resolution) volumes unless a sufficiently high number of PCA components are analyzed, which is not practically feasible.

Consequently, PCA analysis of cryoEM data should be considered as a way to describe large conformational changes, but not as a path to study more detailed macromolecular motions at the level of atomic or secondary structure.

## 3. PCA components should be analyzed collectively   

In cryoEM, it is customary to interpret each principal component independently. [For instance, the first principal component may represent a rotation of a certain part of the macromolecule, the second principal component a shift of another part *etc.*; see Figs. 2, 4 and 5 in Punjani & Fleet (2020 ▸); the reader should note that this is not a criticism of that specific work, as its authors are fully aware of the importance of the distribution of images in the PCA subspace. This interpretation of the isolated bases is common in other structural studies (Chiduza *et al.*, 2019 ▸)]. However, in the light of the analysis in the previous section, PCA provides only a basis in which the deformed volumes can be expressed. The important information is in the combination of the decomposition coefficients, *c*_*n*_, and the volume basis, *V*_*n*_; that is, at the level of volume and not at the level of coefficients or eigenvolumes alone. Stated differently, the interpretation of the eigenvolumes alone does not necessarily follow any ‘biological feature’, but the combined set of eigenvolumes and coefficients is the set that allows a compact analysis of our data set: it is like interpreting the waves of the Fourier transform basis, only these ‘waves’ are specially adapted to the shape and correlation of the underlying macromolecules.

## 4. A simple example   

**Figure 1:**
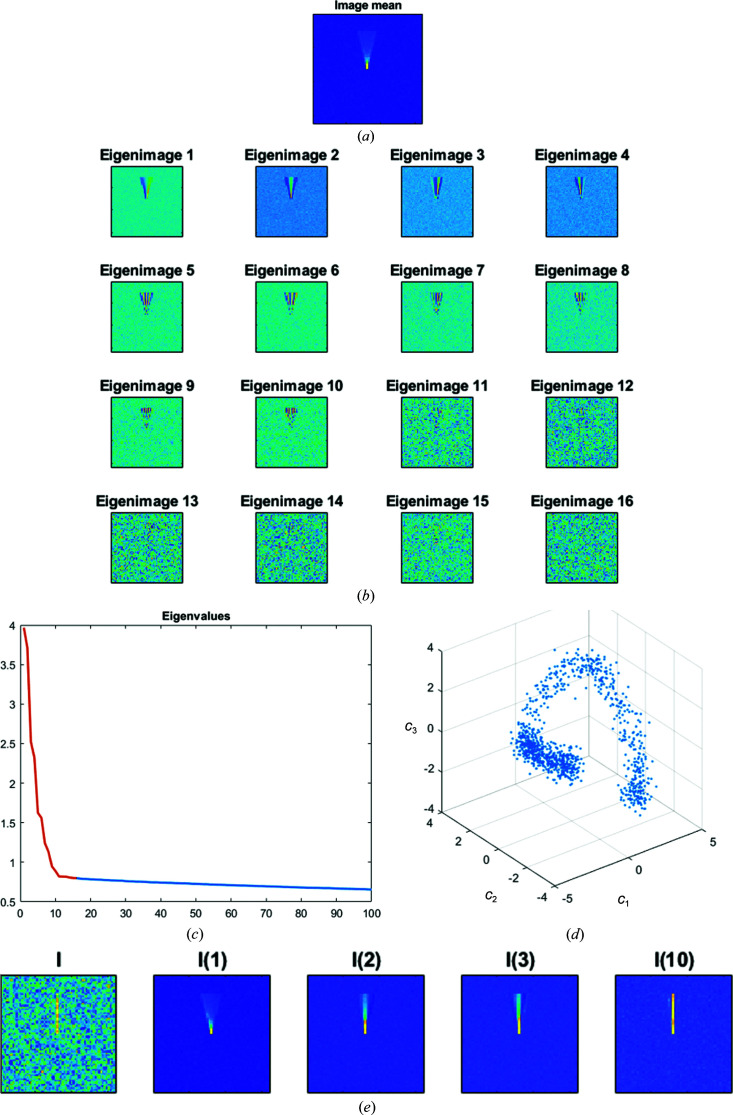
Example of PCA analysis of a small detail moving in an image. The figure shows (*a*) the mean of 1000 images, (*b*) the 16 first eigenimages, (*c*) a plot of eigenvalues, (*d*) a projection of the input data set onto the first three principal components and (*e*) a sample image and its reconstruction with one, two, three and ten principal components.

To illustrate all of these ideas, we have performed a simulated example in which a thin line (an idealized representation of an α-helix) randomly rotates ±15° around a point situated in the middle of the image (for simplicity of representation the example is 2D, but the same ideas apply in 3D), with noise added to the image. We simulated 1000 of these images. In Fig. 1 ▸ we show the mean of the input data set, the corresponding eigenimages (PCA basis), the plot of eigenvalues, the representation of each of the 1000 images in (*c*_1_, *c*_2_, *c*_3_) space and one of the images with its reconstruction using one, two, three and ten eigenimages of the basis.

It can be seen that (i) eigenimages 1–10 have increasing frequency content (as expected from the harmonic functional analysis), (ii) from eigenimage 11 it is challenging to visualize any structural detail (as expected from the difficulty in estimating PCA components in noisy environments), (iii) at least ten coefficients are needed to obtain a meaningful representation of the input images, (iv) a trajectory of images is clearly seen in (*c*_1_, *c*_2_, *c*_3_) space, meaning that understanding of the input images cannot be obtained solely based in terms of the eigenimages, and (v) the reconstructions with a small number of eigenimages show very low-resolution details that hinder the understanding of the underlying deformation.

## 5. Conclusions   

The analysis of the volume covariance matrix and the principal components is effectively connected to the continuous flexibility problem encountered in cryoEM, and it has successfully been used in several previous experimental examples (Melero *et al.*, 2020 ▸).

However, we must understand its limits; in particular, its limitation to describing movements at the level of atomic or secondary-structure details. Actually, there is a trade-off between the extent of the movement and the size of the object being moved. In this way, small objects that move short distances can be safely analyzed with PCA to high resolution. On the other hand, it may be an excellent tool to describe the movement of whole domains or large movements of large portions of the macromolecule. Additionally, the PCA description of the movement must be analyzed at the level of reconstructed volumes combining the different elements of the bases and not at the level of individual coefficients or eigenvolumes alone.
